# Data on a Laves phase intermetallic matrix composite in situ toughened by ductile precipitates

**DOI:** 10.1016/j.dib.2017.08.005

**Published:** 2017-08-03

**Authors:** Alexander J. Knowles, Ayan Bhowmik, Surajit Purkayastha, Nicholas G. Jones, Finn Giuliani, William J. Clegg, David Dye, Howard J. Stone

**Affiliations:** a Department of Materials Science and Metallurgy, University of Cambridge, Cambridge CB3 0F3, UK; b Department of Materials, Imperial College, South Kensington, London SW7 2AZ, UK

## Abstract

The data presented in this article are related to the research article entitled “Laves phase intermetallic matrix composite in situ toughened by ductile precipitates” (Knowles et al.) [1]. The composite comprised a Fe_2_(Mo, Ti) matrix with bcc (Mo, Ti) precipitated laths produced in situ by an aging heat treatment, which was shown to confer a toughening effect (Knowles et al.) [1]. Here, details are given on a focused ion beam (FIB) slice and view experiment performed on the composite so as to determine that the 3D morphology of the bcc (Mo, Ti) precipitates were laths rather than needles. Scanning transmission electron microscopy (S(TEM)) micrographs of the microstructure as well as energy dispersive X-ray spectroscopy (EDX) maps are presented that identify the elemental partitioning between the C14 Laves matrix and the bcc laths, with Mo rejected from the matrix into laths. A TEM selected area diffraction pattern (SADP) and key is provided that was used to validate the orientation relation between the matrix and laths identified in (Knowles et al.) [1] along with details of the transformation matrix determined.

**Specifications Table**

**Table:** 

Subject area	*Materials Science*
More specific subject area	*Metallurgy, Titanium Alloys*
Type of data	*Images (microscopy: FIB-SEM, STEM, STEM-EDX, TEM SADP)*
How data was acquired	*FIB-SEM FEI Helios, TEM JEOL 2100F*
Data format	*Analysed*
Experimental factors	*Arc melted Fe-30Ti-10Mo (at.%) aged at 1000°C for 500 h*
Experimental features	*Fe*_*2*_*(Mo, Ti) C14 Laves matrix composite with bcc (Mo, Ti) precipitated laths*
Data source location	*Department of Materials Science and Metallurgy, University of Cambridge, Cambridge, CB3 0F3, UK*
	*Department of Materials, Imperial College, South Kensington, London SW7 2AZ, UK*
Data accessibility	*Data is with this article and*[1]

**Value of the data**

• Determination of the 3D microstructure of a new Laves phase intermetallic matrix composite through a focused ion beam (FIB) slice and view experiment and surface reconstruction.
• STEM micrographs and STEM-EDX mapping identifying the elemental partitioning between C14 Laves matrix and bcc (Mo, Ti) precipitated laths.
• Details of the determination and validation of the orientation relation between matrix and laths identified in [1].

## 1 Data

Here we report data obtained from a Fe-30Ti-10Mo (at%) alloy [1]. This alloy was selected to explore the toughening effect of A2 (Mo, Ti) precipitates produced within a Laves phase intermetallic matrix composite during heat treatment. Here, we present the microstructure in 3D from a focused ion beam (FIB) slice and view experiment, as well as scanning transmission electron microscopy (S(TEM)) micrographs, energy dispersive X-ray spectroscopy (EDX) maps, a TEM selected area diffraction pattern (SADP) and details of the transformation matrix determination provided in [1].

## 2 Experimental design, materials and methods

Full details of the preparation of the alloy are provided in [1]. The Fe-30Ti-10Mo (at%) alloy was produced by arc melting >99.9 at% purity elements under an argon atmosphere. Sections of the ingot were encapsulated within quartz ampoules under argon and aged at 1000 °C for 500 h, followed by water quenching.

An FEI Helios NanoLab 600 dual beam FIB-scanning electron microscopy (SEM) system was used to perform a slice and view experiment through a ~15×15×15 µm volume by sequential FIB slicing followed by collection of secondary electron (SE) micrographs. The SE micrographs were then binarised and segmented using ImageJ to separate the C14 and A2 (bcc) phases, after which the images were stacked and an iso-surface produced using the Avizo software package.

Electron transparent foils for transmission electron microscopy (TEM) were produced by electro-polishing using a solution of 10 vol% perchloric acid in methanol at −30 °C and 18 V, followed by final polishing using a GATAN precision ion polishing system operated at 5 kV, as in [1]. TEM and scanning TEM (STEM) were carried out using JEOL 2100F and FEI Osiris microscopes respectively.

**Fig. 1:**
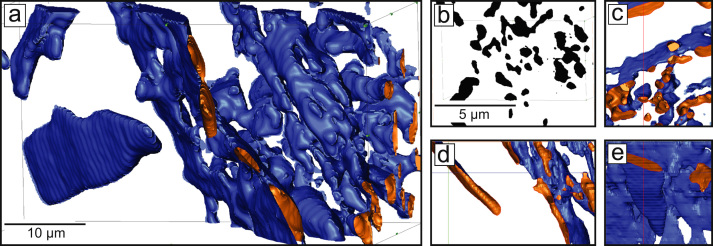
Heat treated alloy FIB-SEM slice and view (a) 3D surface reconstruction of the A2 phase, (b) example binarised FIB-SEM slice, as well as slices through the A2 phase surfaces in the (c) *x*–*y*, (d) *x*–*z*, and (e) *x*–*z* planes.

From SEM micrographs of the aged alloy characterised in [1] uncertainty existed as to whether the precipitated A2 phase occurred as laths or as needles. In order to resolve this, a FIB slice and view experiment was performed, an example binarised SEM SE micrograph from which is shown in Fig. 1b. The 3D surface reconstruction of the laths in the Fe60Ti30Mo10 heat treated alloy can be seen in Fig. 1 along with *x*–*y*, *x*–*z* and *y*–*z* slices of the reconstruction. From this, it was identified that the A2 phase does occur as laths and not as needles [1].

**Fig. 2:**
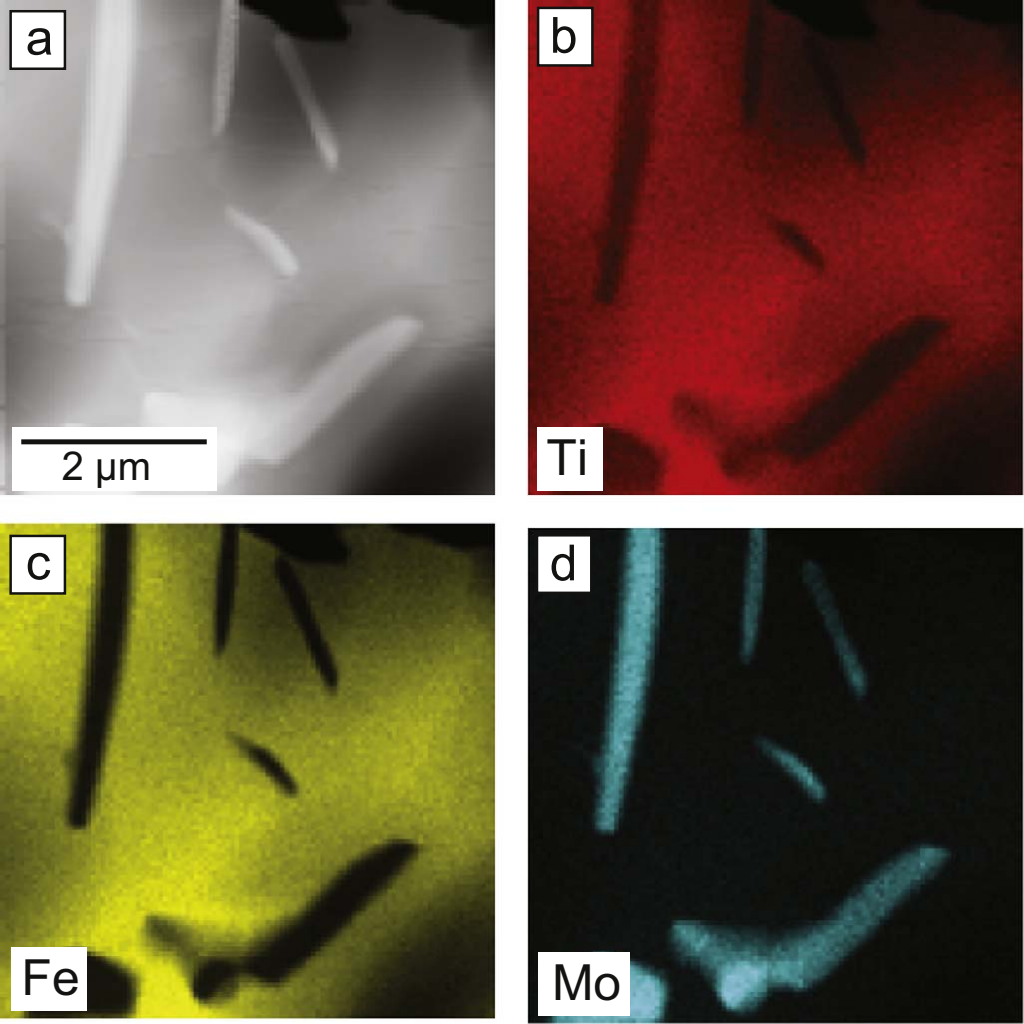
Heat treated alloy (a) HAADF-STEM image, as well as STEM-EDX maps of (b) Ti, (c) Fe and (d) Mo.

In order to study the chemical segregation between the fine scale A2 plates and the C14 matrix STEM-EDX was employed, Fig. 2. The high-angle annular dark-field (HAADF)-STEM micrograph in Fig. 2a showed phase contrast similar to that observed in SEM imaging [1], with the C14 matrix appearing dark and the A2 plates bright according to their relative average Z. STEM-EDX maps for Fe, Ti and Mo, Fig. 2b–d respectively, demonstrated the segregation of elements between the two phases, with the A2 laths being richer in Mo and leaner in Fe and Ti than the C14 matrix. This was consistent with point analyses of the two phases [2], where the isolated laths and those within the A2-C14 two-phase interpenetrating network were found to have similar compositions.

**Fig. 3:**
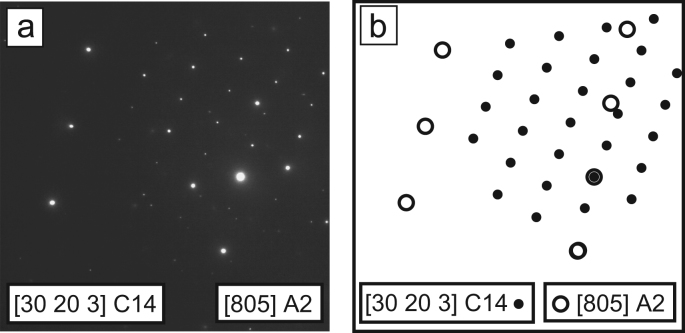
Heat treated alloy (a) SADP of [805]_A2_ and [30 20 3]_C14_ and (b) corresponding key.

A TEM SADP and key of [805]_A2_ and [30 20 3]_C14_ is shown in Fig. 3 that was used to validate the orientation relation and transformation matrix between the matrix and laths identified in [1]. Further details of the determination of the transformation matrix are included in the Appendix.

## Appendix

The parallelism described by Eq. (1) in [1] can be expressed in terms of column vectors, representing crystal directions, as:

(A1) $$\begin{array}{c} a_{C14}\\ a_{C14}\\ c_{C14} \end{array}\left[\begin{array}{ccc} 1 & 0 & 0\\ 0 & 1 & 0\\ 0 & 0 & 1 \end{array}\right]n_{C14}=\begin{array}{c} a_{A2}\\ a_{A2}\\ a_{A2} \end{array}\left[\begin{array}{ccc} \frac{1}{\sqrt{11}} & \frac{1}{\sqrt{11}} & \frac{3}{\sqrt{11}}\\ \frac{1}{\sqrt{3}} & \bar{\frac{1}{\sqrt{3}}} & \bar{\frac{1}{\sqrt{3}}}\\ \frac{1}{\sqrt{6}} & \frac{2}{\sqrt{6}} & \bar{\frac{1}{\sqrt{6}}} \end{array}\right]n_{A2}$$

Given the orientation relationship described by Eq. (1) in [1], we can determine the transformation matrix _A2_*T*_C14_ relating the unit vectors, in the two phases, ***n***_A2_ and ***n***_C14_, which are the column vectors:

(A2) $$n_{A2}{=}_{A2}T_{C14}n_{C14}$$

After transposing and substituting the values of *a*_A2_ and *a*_C14_, Eq. (A1) can be expressed in form of Eq. (A2) as follows:

(A3) $$n_{A2}=\left[\begin{array}{ccc} 0.1976 & 0.3789 & 0.1644\\ 0.1976 & -0.3789 & 0.3287\\ 0.5936 & -0.3789 & -0.1644 \end{array}\right]n_{C14}$$

Converting the 3×3 matrix into an orthogonal matrix, _A2_T_C14_ (Eq. (2) in [1]), the relationship can be rewritten as:

(A4) $$n_{A2}=\left[\begin{array}{ccc} 0.3015 & 0.5774 & 0.4082\\ 0.3015 & -0.5774 & 0.8164\\ 0.9045 & -0.5774 & -0.4082 \end{array}\right]n_{C14}$$
